# Application of H_2_N-Fe_3_O_4_ Nanoparticles for Prostate Cancer Magnetic Resonance Imaging in an Animal Model

**DOI:** 10.3390/ijms251910334

**Published:** 2024-09-26

**Authors:** Barbara Blasiak, David MacDonald, Krzysztof Jasiński, Fong-Yu Cheng, Boguslaw Tomanek

**Affiliations:** 1 The Henryk Niewodniczanski Institute of Nuclear Physics Polish Academy of Sciences, Radzikowskiego 152, 31-342 Krakow, Poland; david.macdonald@ifj.edu.pl (D.M.); krzysztof.jasinski@ifj.edu.pl (K.J.); tomanek@ualberta.ca (B.T.); 2 Department of Chemistry, Chinese Culture University, Taipei 11114, Taiwan; zfy3@ulive.pccu.edu.tw; 3 Division of Medical Physics, Department of Oncology, University of Alberta, 8303 112 St. NW, Edmonton, AB T6G 2T4, Canada

**Keywords:** magnetic resonance imaging (MRI), contrast agents, nanoparticles, prostate cancer, mouse animal model, iron oxide nanoparticles

## Abstract

This paper presents the efficacy of a contrast agent based on H_2_N-Fe_3_O_4_ nanoparticles for the detection of prostate cancer in an animal model using a preclinical 9.4 T MRI system. The relaxivities r_1_ and r_2_ of the nanoparticles were 6.31 mM^−1^s^−1^ and 8.33 mM^−1^s^−1^, respectively. Nanoparticles injected in a concentration of 2 mg Fe/mL decreased the tumor-relative T_1_ relaxation across all animals from 100 to 76 ± 26, 85 ± 27, 89 ± 20, and 97 ± 16 12 min 1 h, 2 h, and 24 h post injection, respectively. The corresponding T_1_ decrease in muscle tissues was 90 ± 20, 94 ± 23, 99 ± 12, and 99 ± 14. The relative T_2_ changes in the tumor were 82 ± 17, 89 ± 19, 97 ± 14, and 99 ± 8 12 min, 1 h, 2 h, and 24 h post injection, respectively, while, for muscle tissues, these values were 95 ± 11, 95 ± 8, 97 ± 6, and 95 ± 10 at the corresponding time points. The differences in the relative T_1_ and T_2_ were only significant 12 min after injection (*p* < 0.05), although a decrease was visible at each time point, but it was statistically insignificant (*p* > 0.05). The results showed the potential application of H_2_N-Fe_3_O_4_ nanoparticles as contrast agents for enhanced prostate cancer MRI.

## 1. Introduction;

Prostate cancer (PC) is a curable disease if diagnosed early; however, if undetected, it can spread aggressively and has few early symptoms, leading to late diagnosis and, hence, problematic outcomes [1]. Therefore, early diagnosis and timely efficient treatment are essential for effective cancer management. Currently, the established standard for PC diagnosis involves prostate-specific antigen testing and digital rectal examination [2]. However, these diagnostic methods often lead to overdiagnosis and overtreatment [3], given that only a small percentage of these cancers metastasize, and approximately 3% of them result in fatalities [3,4,5,6,7]. While transrectal ultrasonography, computed tomography (CT), positron emission tomography (PET) [8], and magnetic resonance imaging (MRI) [9,10,11] are utilized for PC diagnosis and staging, their limited value stems from their low sensitivity (CT, US), specificity (PET), tumor contrast (CT), and image resolution (PET) in detecting tumor tissues and lymph node metastases [12]. These methods, in particular MRI, providing superior soft-tissue contrast, are enhanced using contrast agents [13]. MRI contrast agents are mostly based on gadolinium [14] or iron oxide [15], and their r_1_ and r_2_ relaxivities decrease with an increasing magnetic field. This fact should be taken into account when considering translation from a high preclinical field (7 T and above) to clinical studies (3 T and below) [16].

Superparamagnetic iron oxide nanoparticles (SPIONSs) are commonly used in MRI as T_2_ contrast agents mostly due to their high r_2_ relaxivity and low toxicity [15]. Most SPIONSs are 3 to 30 nm in size [17] and can be coated with biocompatible materials, which can further reduce the overall toxicity [18]. SPIONSs can be functionalized with antibodies or peptides, allowing binding to specific targets, providing a high specificity. They can be modified to alter their size, shape, and coating properties. For example, Tse et al. conjugated iron oxide nanoparticles (NPs) with the J591 antibody for targeting the prostate-specific membrane antigen (PSMA), showing enhanced contrast accumulation in prostate cancer in preclinical MRI [19]; meanwhile, Zhu et al. showed similar results with polypeptide-labeled SPIONSs [20]. Targeted SPIONSs enhance some tumors, but not all prostate tumors express PSMAs [17].

While SPIONSs predominantly reduce the T_2_ relaxation time, causing the so-called “negative” tumor contrast in T_2_-weigthed images, Gd-based contrasts reduce mostly T_1_, providing positive contrast in T_1_-weighted MRI [21]. The NPs are internalized by macrophages and excreted by the kidneys. Their impact on the cancer image contrast depends on the size, concentration, and type of NPs, as well the magnetic field strength [22]. NPs are applied naked or synthesized with vehicles such as peptides [23] or specific antibodies [24] for targeted delivery. Clinically approved MRI contrast agents, such as Gd-DTPA, have an r_1_ relaxivity of 4.79 mM^−1^s^−1^ and an r_2_ of 5.14 mM^−1^s^−1^ at 1.5T and an r_1_ of 4.05 mM^−1^s^−1^ and an r_2_ of 5.09 mM^−1^s^−1^ at 3 T [25]. A study by Fan Pu et al. showed the gadolinium-based contrast agent relaxivities r_1_ and r_2_ to be 18.6 mM^−1^s^−1^ and 94 mM^−1^s^−1^, respectively, at 7T [26]. Hagberg et al. carried out a similar study with Gd-based NPs at 9.4 T and found the r_1_ and r_2_ to be 18 mM^−1^ s^−1^ and 21.6 mM^−1^s^−1^ [27]. To increase specificity, NPs are often synthesized with lipids or other delivery vehicles, which impact the relaxivity. For example, the r_1_ relaxivity of Gd-CP027 bound to albumin was found to be 48 mM^−1^·s^−1^at 0.5 T, 31 mM^−1^s^−1^ at 1.5 T, and 9.4 mM^−1^s^−1^ at 9.4 T, allowing one to generate a visible signal change at a local concentration of about 0.1 mM [28]. Studies of SPIONSs at 7 T showed r_1_ and r_2_ of 3.4 mM^−1^s^−1^ and 60 mM^−1^s^−1^, respectively [22], while other groups reported the r_1_ and r_2_ values to be 5.6 mM^−1^s^−1^ and 72 mM^−1^s^−1^ at 9.4 T [29]. A much higher relaxivity was shown for NPs composed of paramagnetic Dy^3+^ and Gd^3+^ (T_2_ and T_1_ contrast agents, respectively) [30], with relaxivities of r_1_ = 20.2 mM^–1^s^–1^ and r_2_ = 32.3 mM^–1^s^–1^ at clinical 3 T and r_1_ = 9.4 mM^–1^s^–1^ and r_2_ = 144.7 mM^–1^s^–1^ at 9.4 T [27]. The summary of the r_1_ and r_2_ values of the selected NPs is provided in Table 1, while detailed data on the various NPs are presented by Pellico et al. [31].

The incorporation of nitrogen into iron- or gadolinium-based contrast agents has shown to alter their magnetic properties, improving their performance as MRI contrast agents [33,34]. SPIONSs can be doped with nitrogen during synthesis, which modifies their electronic structure and increases their effective magnetic moment, leading to improved MRI contrast. Additionally, nitrogen-doped SPIONSs may exhibit improved biocompatibility and stability compared to their undoped counterparts [35]. Nitrogen can also stabilize gadolinium ions and enhance the biocompatibility of gadolinium-based contrast agents; it also affects their pharmacokinetics and biodistribution. Nitrogen-containing ligands are commonly used to incorporate gadolinium ions into contrast agents. These ligands are designed to form stable complexes with gadolinium ions, allowing for their controlled release and the enhancement of contrast in MR images [33]. Furthermore, recent studies showed that the integration of H_2_N with Fe_3_O_4_ NPs led to higher relaxation rates (R_1_ = 1/T_1_) than conventional Fe_3_O_4_-based nanoparticles. This provided high in vitro biocompatibility [30]. H_2_N-Fe_3_O_4_ NPs were also used as magnetic carriers for nucleotides and enzyme manipulation through electrostatic interactions due to their positively charged surface [35,36,37,38].

While the synthesis method and analysis of H_2_N-Fe_3_O_4_ NPs have already been provided by Shieh et al. [37], this paper reports the application of H_2_N-Fe_3_O_4_ NPs for the enhanced detection of prostate cancer in a nude mouse model using a preclinical MRI system operating at 9.4 T. The results showed that the NPs enabled the improved detection of prostate cancer in the animal model compared to previous studies.

## 2. Results

**Figure 1:**
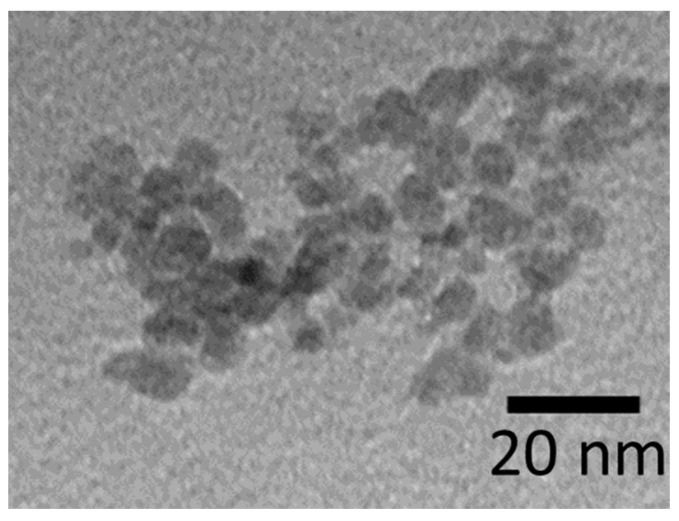
Transmission electron microscopy image of the H_2_N-Fe_3_O_4_ NPs.

**Figure 2:**
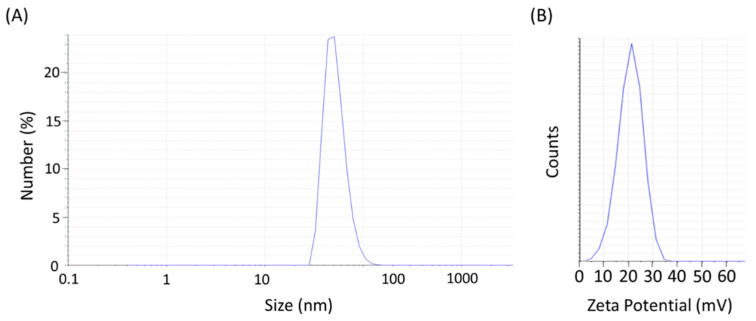
(**A**) Hydrodynamic diameter and (**B**) zeta potential of the H_2_N-Fe_3_O_4_ nanoparticles.

The images of the NPs obtained with the TEM revealed that the diameter of the H_2_N-Fe_3_O_4_ NPs was around 7.4 nm (Figure 1). Figure 2A shows the hydrodynamic diameter of the H_2_N-Fe_3_O_4_ NPs, which is around 17 nm. The surface charge of the H_2_N-Fe_3_O_4_ NPs was +20.8 mV (Figure 2B).

**Figure 3:**
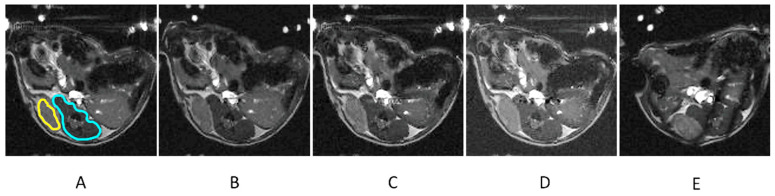
T_1_-weighted MR images of a mouse with prostate cancer before the injection (**A**) and 12 min (**B**), 1 h (**C**), 2 h (**D**), and 24 h (**E**) after the injection of the H_2_N-Fe_3_O_4_ NP contrast agent. The regions of interest encompassing the tumor (T) and muscles (M) have been manually selected and marked (in (**A**) only for clarity) with yellow and blue color, respectively. The post-injection images show increased signals from the tumor 12 min, 1h, and 2h after the injection and a return to the initial value 24 h after the injection. Signals from other organs (e.g., intestines) remain the same at each time point, except for 24 h after the injection due to the repositioning of the animals. The visible changes in the signal intensity due to the contrast injections causing the T_1_ relaxation decrease in both cancer and muscle tissues are quantified and analyzed below.

Figure 3 shows the T_1_-weighted MRI of a mouse with PC pre injection (A) and 12 min (B), 1 h (C), 2 h (D), and 24 h post injection (E) of H_2_N-Fe_3_O_4_ NPs at a concentration of 2 mgFe/mL.

**Figure 4:**
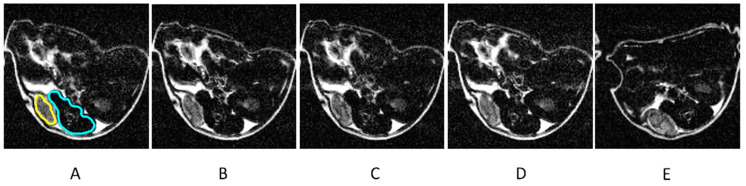
T_2_-weighted magnetic resonance (MR) images of a mouse with prostate cancer before the injection (**A**) and 12 min (**B**), 1h (**C**), 2h (**D**), and 24 h (**E**) after the injection of the H_2_N-Fe_3_O_4_ NP contrast agent. The regions of interest encompassing the tumor (T) and muscles (Ms) have been manually selected and marked (in (**A**) only for clarity) with yellow and blue color, respectively. The post-injection images show decreased signals from the tumor 12 min, 1 h, and 2 h after the injection and a return to the initial value 24 h after the injection. Signals from other organs (e.g., intestines) remain the same at each time point, except for 24 h after the injection due to the repositioning of the animals. The visible changes in the signal intensity due to the contrast injections causing T_2_ relaxation decrease are quantified and analyzed below.

Figure 4 shows the T_2_-weighted MRI of a mouse with PC pre injection (A) and 12 min (B), 1 h (C), 2 h (D), and 24 h post injection (E) of H_2_N-Fe_3_O_4_ NPs at a concentration of 2 mgFe/mL.

Because the T_1_ and T_2_ relaxation values varied between the animals prior to injection, we normalized the changes by calculating the relative change (RC) as follows:

(1) $$\mathrm{RC}=\left(\frac{{\;\bar{T}}_{1,2}\left(t\right)}{{\;\bar{T}}_{1,2}\left(0\right)}\right)\times 100,$$

where ${\bar{T}}_{1,2}\left(0\right)$ and ${\bar{T}}_{1,2}\left(t\right)$ are the averaged T_1_ and T_2_ relaxation times across all animals at times 0 and t, respectively. The RC was calculated for both the PC and muscles.

**Figure 5:**
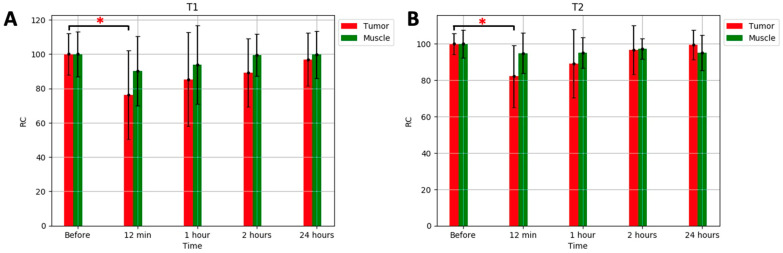
The RC values over 6 animals for the T_1_ (**A**) and T_2_ (**B**) relative relaxation within the tumor and muscles before, 12 min, 1 h, 2 h, and 24 h after the injection. The data show a statistically significant (*p* < 0.05) difference (indicated with *) in the relative change (RC) of T_1_ and T_2_ 12 min after the injection. The decrease 1 h, 2 h, and 24 h after the injection was visible but not statistically significant (*p* > 0.05).

Figure 5 shows the RC values over all the animals for T_1_ and T_2_ within the tumor and muscles before, 12 min, 1 h, 2 h, and 24 h after the injection.

Figure 5 shows the changes in the T_1_ and T_2_ RC values due to contrast accumulation in the tumor and muscles across all animals. The T_1_ RC (Figure 5A) of the tumor decreased from 100 to 76 ± 26 (*p* = 0.038), 85 ± 27 (*p* = 0.12), and 89 ± 20 (*p* = 0.078) 12 min, 1 h, and 2 h post injection, respectively, across all the animals. The muscle tissues exhibited decreases to 90 ± 20 (*p* = 0.15), 94 ± 23 (*p* = 0.27), and 99 ± 12 (*p* = 0.44) 12 min, 1 h, and 2 h post injection, respectively, and were not significant (*p* > 0.05). After 24 h, the T_1_ of both the tumor and muscles returned to the pre-injection value (97 ± 16 (*p* = 0.14) and 100 ± 14 (*p* = 0.46), respectively).

Figure 5B shows the T_2_ RC value changes in the tumor and muscles. The T_2_ RC of the tumor decreased from 100 to 82 ± 17 (*p* = 0.028), 89 ± 19 (*p* = 0.12), and 97 ± 14 (*p* = 0.30) 12 min, 1 h, and 2 h post injection, respectively, across all the animals. The muscle tissues exhibited decreases to 95 ± 11 (*p* = 0.059), 95 ± 8 (*p* = 0.75), and 98 ± 6 (*p* = 0.17) 12 min, 1 h, and 2 h post injection, respectively, and were not significant (*p* > 0.05). After 24 h, the T_2_ RC of both tumor and muscles returned to the pre-injection value (99 ± 8 (*p* = 0.46) and 95 ± 10 (*p* = 0.12), respectively).

The changes in the tumor T_1_ and T_2_ RC values were only significant (*p* < 0.05) 12 min after the injection.

## 3. Discussion

The obtained results showed a significant reduction in the T_1_ RC within the prostate tumor due to the administration of the H_2_N-Fe_3_O_4_ NP contrast agent 12 min post injection. The tumor T_1_ RC decreased from 100 to 76 (*p* < 0.05), and a decrease from 100 to 90 (*p* > 0.05) was observed in the muscle tissue, while the T_2_ tumor RC decreased from 100 to 82 (*p* < 0.05), with muscle tissue decreasing from 100 to 95 (*p* > 0.05). The changes in the RC times within the muscle tissue were less pronounced than those observed in the tumor region. The difference suggests a higher accumulation of the contrast agent in the tumor than in the muscle tissue. These findings show the efficacy of the H_2_N-Fe_3_O_4_ NP contrast agent, demonstrating its pronounced impact on both tumor and muscle tissues, with a notably greater effect observed on the tumor tissue.

As the applied NPs were non-selective, they accumulated in both the tumor and muscles. However, the decrease in the RC in the tumor was higher at each time point after their administration due to the enhanced permeability and retention (EPR) effect of the tumor. The relaxation values returned to the pre-injection values 24 h post injection in the tumor and muscles due to wash out. The toxicity profile of the H_2_N-Fe_3_O_4_ NP contrast agents had been investigated in prior studies and had been found to be low [32], showing potential as a safe contrast agent. A capping agent (glycine) may contribute to the enhanced biocompatibility and suitability of Fe_3_O_4_ for biomedical applications [32].

The greatest differences in the RC relaxation times and, hence, in the accumulation of nanoparticles in tumor and muscles are visible 12 min after administration. This is likely related to a more extensive vasculature within the tumor than in the muscles.

The accurate delineation of the prostate cancer boundary is of utmost importance for successful treatment [38]. To achieve sufficient contrast enhancement, most of the current contrast agents used for cancer imaging include gadolinium-based agents [13,39]. While these agents are widely utilized, they do not always provide optimal contrast enhancement for prostate cancer detection, as rapid renal clearance reduces their diagnostic capability. However, their accumulation may be further enhanced by conjugating NPs with other vehicles such as antibodies, providing potential to increase the diagnostic capabilities of prostate cancer MRI. An alternative to Gd-based NPs are SPIONSs due to their low toxicity. Furthermore, macrophages in cancerous tissues, e.g., prostate cancer, are able to internalize SPIOs, leading to their accumulation and, consequently, the desired decrease in signal in T_2_-weighted magnetic resonance (MR) images. Various coatings (e.g., dextran) and hydrodynamic diameters (30-3000nm) of iron oxide NPs have been investigated as MRI contrast agent for many cancers. Their r_1_ vary from 10 to 15 mM^−1^s^−1^ and their r_2_ from 30 to 89 mM^−1^s^−1^ at 1.5T [40]. The relaxivities r_1_ and r_2_ of the H_2_N-Fe_3_O_4_ NPs were shown to be 6.3 mM^−1^s^−1^ and 8.33 mM^−1^s^−1^ at 9.4 T [37], with a significantly lower r_2_ than Resovit^®^ at 9.4 T. Kader et al. [15] investigated the application of ferumoxytol (iron oxide coated with carboxylmethyl-dextran) with a core size of 3 nm to 12 nm for prostate cancer detection using a xenograft mouse model. A significant decrease in signal intensity between the pre- and 24 h post- contrast images (n = 14; *p* < 0.001) was detected [15]. In human studies, ferumoxtran-10 (with dextran coating) was used to detect prostate cancer metastases in lymph nodes. MRI was performed before and 24 h after the intravenous injection of 2.6 mg/kg of ferumoxtran-10 in patients with presurgical prostate cancer who underwent lymph node resection or biopsy. The ferumoxtran-10-enhanced MR images identified all patients with nodal metastases [41,42].

The efficacy of NPs in prostate cancer detection, Gd- or iron-based, may be improved by constructing contrast agents that target PSMA using peptides and antigens [19,20,43,44]. Studies in preclinical models have shown a reduction in the T_2_ signal from 2 h to 12 h after the injection of the targeted contrast agents [19,45].

## 4. Materials and Methods

### 4.1. Synthesis of H_2_N-Fe_3_O_4_ Nanoparticles

**Figure 6:**
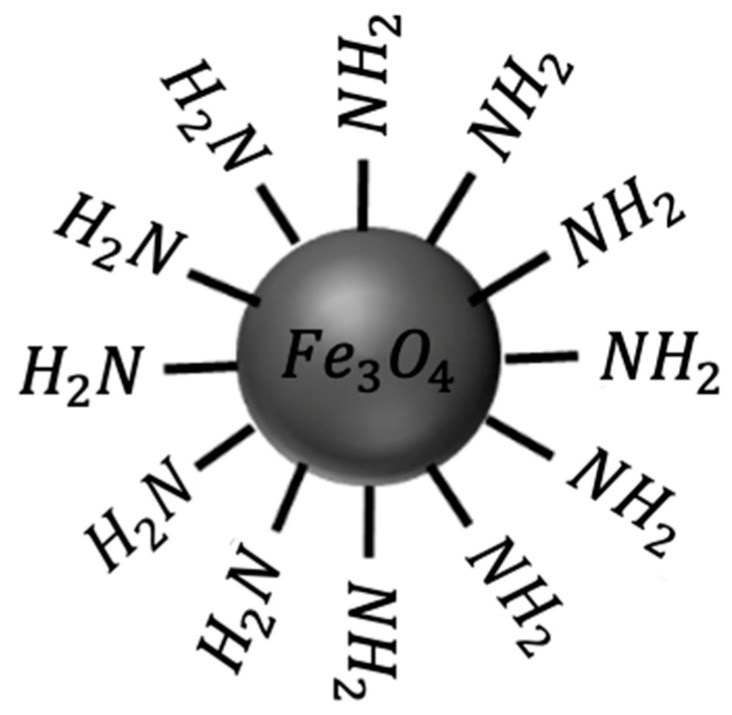
A schematic illustration of the H_2_N-Fe_3_O_4_ nanoparticle.

A co-precipitation process, using covalently bound alginates, was applied to synthesize H_2_N-Fe_3_O_4_ NPs. Details for the synthesis of H_2_N-Fe_3_O_4_ NPs were provided by Shieh et al. [32]. Briefly, to synthesize H_2_N-Fe_3_O_4_ NPs, 2.0 M FeCl_2_ (2.0 mL) and 1.0 M FeCl_3_ (8.0 mL) were mixed with glycine. After 5 min of stirring, a 5 M NaOH solution was gradually added until the solution changed color from yellow to black. The resulting precipitates were collected using a permanent magnet and treated with 3.5 g of glycine dissolved in 30 mL of deionized water, followed by 20 min of sonication. Post sonication, deionized water and acetone were added, and the solution was centrifuged at 10,000 rpm for 10 min. The final precipitates were redispersed in deionized water to obtain a solution of H_2_N-Fe_3_O_4_ NPs, and their iron ion concentration was analyzed with an inductively coupled plasma atomic emission spectroscopy system (CP-AES, Thermo-Element XR, Fisher Scientific Inc., Waltham, MA, USA). A transmission electron microscope (TEM, JEM-1200EX, JOL Ltd., Tokyo, Japan) was used for NP imaging. The zeta potential and hydrodynamic diameter were measured using dynamic light scattering (DLS, ZS90, Malvern Panalytical, Malvern, UK). An illustration depicting an NP is shown in Figure 6.

### 4.2. Animal Model

Six five-week-old male athymic nude mice (Crl: Nu(NCr)-Foxn1nu; CR Strain code: 490, Charles River) were inoculated subcutaneously on their flank with a suspension of 3 × 10^6^ PC3 cells in 50:50 Matrigel (Fisher Scientific, Poznań, Poland) and RPMI-1640 media supplemented with 10% fetal bovine serum and 1% antibiotics/antimycotics. Once the tumors reached a size of about 5 mm in diameter (~3 weeks), the animals were injected via the tail vein with the contrast agent and imaged with MRI. Previous studies showed a very low toxicity for H_2_N-Fe_3_O_4_ NPs [37]. The authors showed that kidney cell viability remained unchanged at dosages from 0.125 to 8 mM after 4 h of exposure. In vitro hemolysis using human blood showed only mild hemolysis (1.3 g/dL) detected at a 0.1 M iron concentration, which is much higher than that required for MR contrast enhancement [32]. The animal experiment was approved by the local Animal Care Committee (permit no. 87/2023).

### 4.3. MRI Experiments

An MRI system equipped with a 9.4 T 21 cm bore magnet (Bruker, Ettlingen, Germany) was used for the in vivo MRI experiments. A 35 mm diameter volume radio frequency coil was positioned over the animal body to encompass the region of interest (ROI), including the tumor. Imaging sessions were conducted before the injection and 12 min, 1 h, 2 h, and 24 h after the injection of 0.25 mL of the H_2_N-Fe_3_O_4_ NP contrast agent with a concentration of 2mg/mL of iron. T_1_- and T_2_- weighted images were collected at each time point.

For the MRI scans, the animals were anesthetized with isoflurane (1.5–2% in 30% oxygen) administered through a nose cone, and they were placed in a cradle positioned within the center of the bore of the magnet. The animals were kept normothermic using a circulating water blanket, and their pulse sequences were triggered with respiration.

For the T_1_- and T_2_-weighted images, inversion recovery with steady-state free precession (IR FISP) and multi-slice multi-echo (MSME) pulse sequences were used. For the T_2_-weighted images, the following pulse sequence parameters were used: 1 mm slice thickness, TE 5 ms, TR 5000 ms, NA 1, 32 echoes, 10 slices, matrix size 128 × 128, and FOV 2.56 cm × 3.0 cm. A single exponential fitting of the echoes was used for the calculation of the T_2_ relaxation times (Bruker, Ettlingen, Germany). For the T_1_ measurements, the IR FISP sequence parameters were the following: TE 1.5 ms, TR 3ms, NA 8, FA 60 deg, number of segments 1, 30 frames, 1 slice, matrix size 128 × 128, FOV 2.56 cm × 3.0 cm, and slice thickness 1 mm. All the results of the changes in the RC relaxation times were processed using a standard analysis of variance (ANOVA); *p* < 0.05 was considered statistically significant.

## 5. Conclusions

The H_2_N-Fe_3_O_4_-based MRI contrast agent significantly reduced both the T_1_ and T_2_ relaxation times in the animal model of PC. These findings show its potential to enhance the imaging contrast for prostate tumors. The observed reductions in the relaxation times highlight the effectiveness of the contrast agent in enhancing tumor detection. The decreases in the T_1_ and T_2_ times within the muscle tissue were less pronounced than in the tumor region, implying a higher concentration of the contrast agent within the tumor volume. The findings show that the presented H_2_N-Fe_3_O_4_-based contrast agent effectively reduced both the T_1_ and T_2_ relaxation times, presenting a promising avenue for improving the accuracy and efficiency of prostate tumor imaging in preclinical applications, as there is a pressing need for an improved PC staging method which enables the more accurate detection of potentially cancerous cells. The lack of a targeting moiety and the lower r_1_ and r_2_ of the NH_2_-Fe_3_O_4_ used in our study compared to the most commonly used contrast agents, such as Resovit^®^, may explain the relatively small changes in the relaxation time of the tumor. Further contrast studies, followed by targeting and clinical applications may allow early tumor detection, enabling curative interventions.

## Figures and Tables

**Table 1:** Relaxivities of the nanoparticles described in this text.

Contrast Agent	r1 [mM^−1^s^−1^]	r2 [mM^−1^s^−1^]	Field Strength [T]	Ref.
Gd-CP027 bound to albumin	48		0.5	[28]
Gd-CP027 bound to albumin	31		1.5	[28]
Gd-CP027 bound to albumin	9.4		9.4	[28]
Gd-DTPA	4.79	5.14	1.5	[25]
Gd-DTPA	4.05	5.09	3	[25]
Gadolinium-based	18.6	94	7	[26]
Gd-based	18	21.6	9.4	[27]
SPIONS	5.6	72	9.4	[29]
Dy/Gd	20.2	32.3	3	[30]
Dy/Gd	9.4	144.7	9.4	[30]
H_2_N-Fe_3_O_4_	6.31	8.33	9.4	[32]
H_2_N-Fe_3_O_4_	6.77	33.56	1.5	[32]
Resovit^®^	1.2	280	9.4	[32]
Resovit^®^	6.7	82	1.5	[32]

## Data Availability

Data is contained within the article.
